# Supplementary material for: InterpolatedXY: a two-step strategy to normalize DNA methylation microarray data avoiding sex bias
Source: Bioinformatics. 2022 Jun 30;38(16):3950–7. doi: 10.1093/bioinformatics/btac436 (PMC9364386; doi:10.1093/bioinformatics/btac436)
Supplement: btac436_Supplementary_Data [file btac436_supplementary_data.pdf]

Supplementary table and figures

Supplementary Table 1: Lists of sample ID used in dataset one and dataset two.

Sample	Sex	Dataset one(n=16)	Dataset two(n=48)
GSM4230892	Female	TRUE	TRUE
GSM4230891	Male	TRUE	TRUE
GSM4230890	Male	TRUE	TRUE
GSM4230889	Female	TRUE	TRUE
GSM4230888	Female	TRUE	TRUE
GSM4230887	Male	TRUE	TRUE
GSM4230886	Male	TRUE	TRUE
GSM4230885	Female	TRUE	TRUE
GSM4230884	Male	TRUE	TRUE
GSM4230883	Male	TRUE	TRUE
GSM4230882	Male	TRUE	TRUE
GSM4230881	Male	TRUE	TRUE
GSM4230880	Male	TRUE	TRUE
GSM4230879	Male	TRUE	TRUE
GSM4230878	Male	TRUE	TRUE
GSM4230877	Male	TRUE	TRUE
GSM4230876	Male	FALSE	TRUE
GSM4230875	Male	FALSE	TRUE
GSM4230874	Female	FALSE	TRUE
GSM4230873	Female	FALSE	TRUE
GSM4230872	Female	FALSE	TRUE
GSM4230871	Female	FALSE	TRUE
GSM4230870	Female	FALSE	TRUE
GSM4230869	Female	FALSE	TRUE
GSM4230868	Female	FALSE	TRUE
GSM4230867	Female	FALSE	TRUE
GSM4230866	Female	FALSE	TRUE
GSM4230865	Female	FALSE	TRUE
GSM4230864	Female	FALSE	TRUE
GSM4230863	Female	FALSE	TRUE
GSM4230862	Female	FALSE	TRUE
GSM4230861	Female	FALSE	TRUE
GSM4230860	Female	FALSE	TRUE
GSM4230859	Female	FALSE	TRUE
GSM4230858	Female	FALSE	TRUE
GSM4230857	Female	FALSE	TRUE
GSM4230856	Female	FALSE	TRUE
GSM4230855	Female	FALSE	TRUE
GSM4230854	Female	FALSE	TRUE
GSM4230853	Male	FALSE	TRUE
GSM4230852	Male	FALSE	TRUE
GSM4230851	Male	FALSE	TRUE
GSM4230850	Male	FALSE	TRUE
GSM4230845	Male	FALSE	TRUE
GSM4230844	Male	FALSE	TRUE
GSM4230843	Male	FALSE	TRUE
GSM4230842	Male	FALSE	TRUE
GSM4230841	Male	FALSE	TRUE

Supplementary Table 2: The fraction of variance explained by sex in the dataset two (n=48) with no normalisation, funnorm normalisation and interpolatedXY adjusted funnorm normalisation.

Fraction of variance explained by sex (%)	raw	funnorm	interpolatedXY adjusted funnorm
Autosomes	6.39	7.05	7.05
X chromosome	93.33	**93.33**	93.21
Y chromosome	97.75	**97.75**	89.12

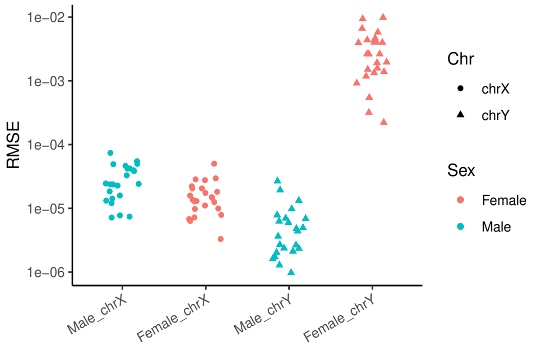

Supplementary Figure 1: Difference between interpolated values and expected values within the adjusted dasen. RMSEs are grouped in four categories: male X chromosomes, female X chromosomes, male Y chromosomes and female Y chromosomes. Dots represent X chromosomes and triangles represent Y chromosomes.

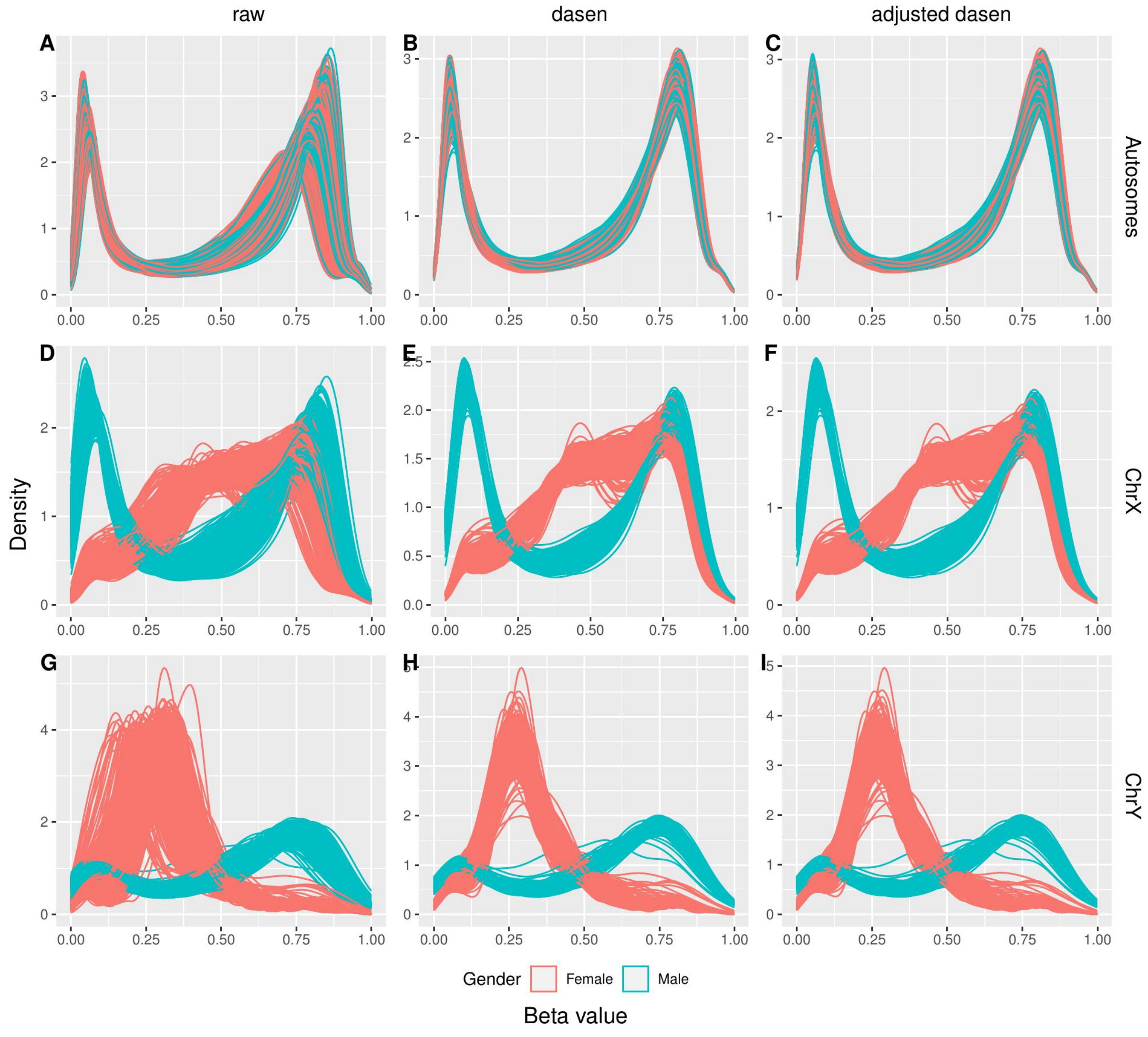

Supplementary Figure 2: Comparisons in methylation beta value density distributions for UKHLS dataset. The three columns illustrate results from raw data (left column), funnorm normalised data (middle column) and the adjusted funnorm normalised data (right column). The three rows show density distributions of autosomal CpGs (first row), X chromosome linked CpGs (second row) and Y chromosome linked CpGs (third row). Red lines represent females and blue lines represent males.

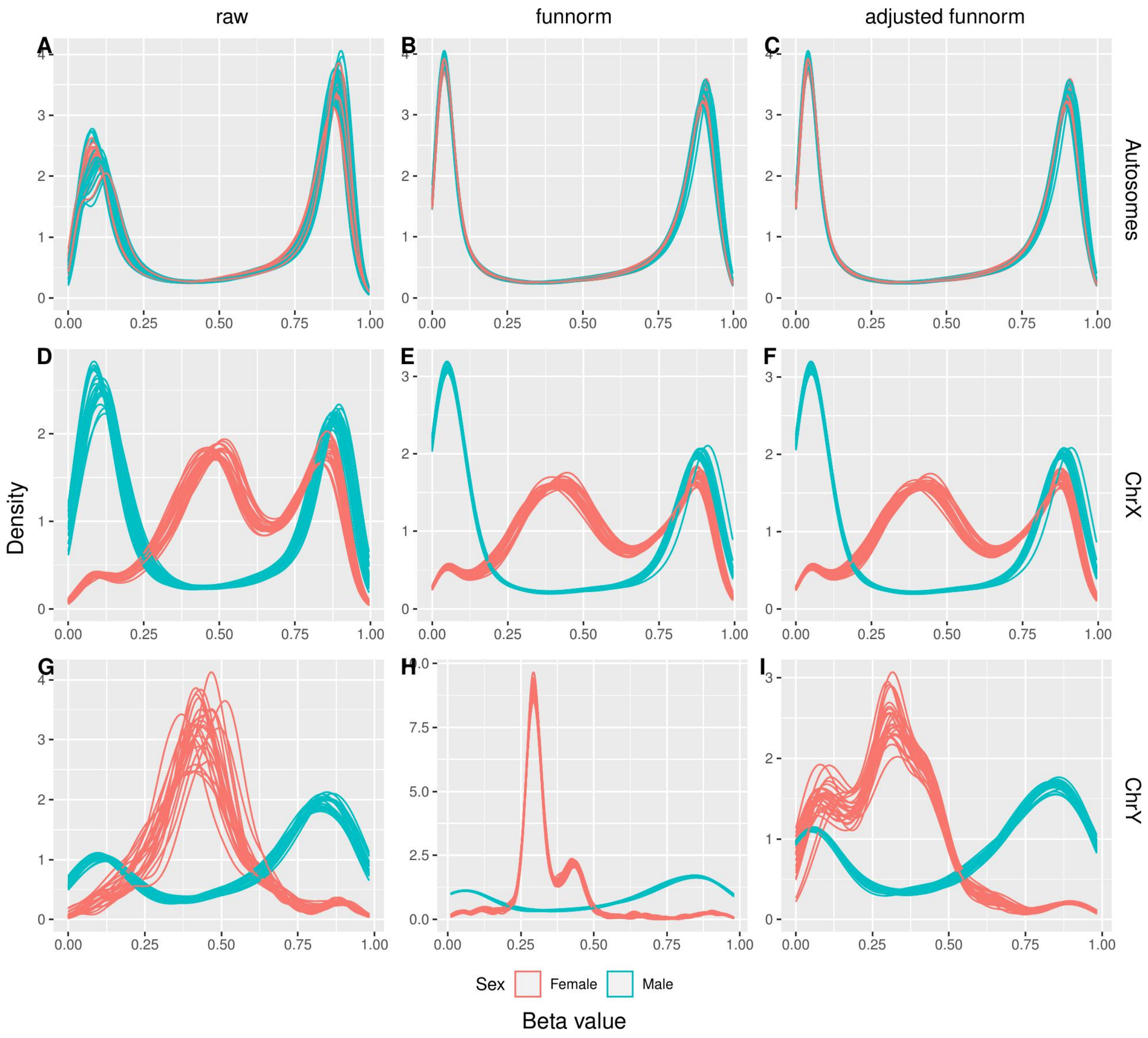

Supplementary Figure 3: Comparisons in methylation beta value density distributions for dataset two. The three columns illustrate results from raw data (left column), normalised data (middle column) and the adjusted funnorm normalised data (right column). The three rows show density distributions of autosomal CpGs (first row), X chromosome linked CpGs (second row) and Y chromosome linked CpGs (third row). Red lines represent females and blue lines represent males.

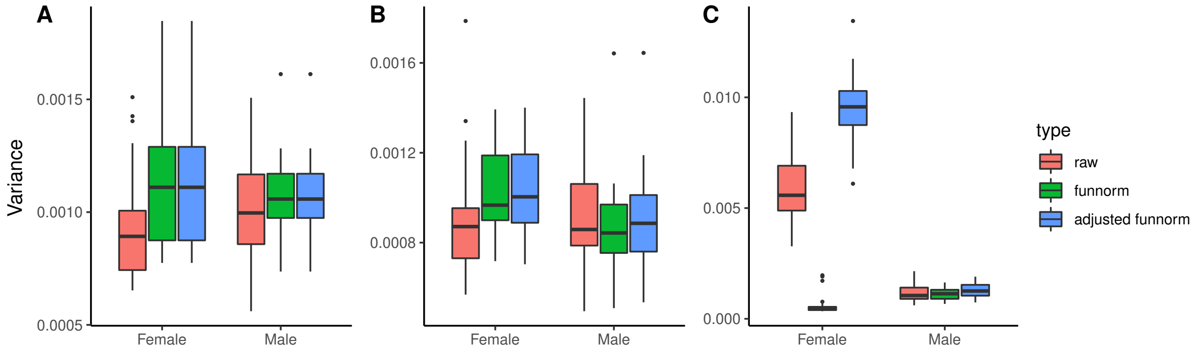

Supplementary Figure 4: Variance comparisons in the dataset two. Boxplots comparing the variance of methylation beta values with three different pre-processing methods (i.e. no normalisation, dasen normalisation and adjusted dasen normalisation) in autosomes (A), X chromosomes (B) and Y chromosomes (C). Females and males are dealt with separately.
